# Introductory Addresses

**Published:** 1851-07

**Authors:** 


### ARTICLE IV.

### INTRODUCTORY ADDRESSES.

A number of these, the flowers of medical literature, have accumulated upon our table, making together quite a *beauquete.*

We will now endeavor to discharge a long neglected duty, by paying our respects to some of them and by laying their characteristics before our readers.

The Introductory of Professor Pallen, at the opening of the last session in the St. Louis University, treats of the influence of reason or science in guiding to the discovery of truth by observation in medicine. It is written in a good style and abounds in happy illustrations from history, with sound inductions from them.

The Introductory of Professor J. R. Allen, of Transylvania University, delivered to a very respectable class assembled to receive instructions in the venerable hall of that, the pioneer Medical School of the West, is encouraging and rebukes the report of the down fall of that school. The lecture dwells upon the discouragements in the way of the physician and handles quackery without gloves.

The Introductory Address of Professor Ware, of Harvard University, is one of the few documents of this kind in which we find a single subject thoroughly and systematically discussed.

The subject here treated is "the Elements of Success in the Medical Profession," and it is handled with a master's hand.

But for the restrictions imposed upon us by the narrowness of our limits, we would give a synopsis of the arguments and illustrations of the author.

The Introductory of Prof. Curran, of Indianapolis, is upon the *position*, *relations* and *duties* of the medical profession. It is well written, and abounds in valuable suggestions, and useful information; but with some of the views expressed we are unable to concur. That the medical profession needs protection by law, we think is an admission of its weakness, which we deny; and if the *people* prefer not to be protected against quackery, we do not see that physicians are responsible for the consequences.

